# Copy number variation and neuropsychiatric illness

**DOI:** 10.1016/j.gde.2021.02.014

**Published:** 2021-06

**Authors:** Elliott Rees, George Kirov

**Affiliations:** MRC Centre for Neuropsychiatric Genetics and Genomics, Division of Psychological Medicine and Clinical Neurosciences, School of Medicine, Cardiff University, Cardiff, United Kingdom

## Abstract

Copy number variants (CNVs) at specific loci have been identified as important risk factors for several neuropsychiatric disorders, such as schizophrenia, autism spectrum disorder, intellectual disability (ID) and depression. These CNVs are individually rare (<0.5% frequency), have high effect sizes, and show pleiotropic effects for multiple neuropsychiatric disorders, which implies a shared aetiology. Neuropsychiatric CNVs are also associated with cognitive impairment and other medical morbidities, such as heart defects and obesity. As most neuropsychiatric CNVs are multigenic, it has been challenging to map their effects onto specific biological processes, although gene-set analyses have implicated genes related to the synapse and chromatin regulation. However, future whole-genome sequencing studies have potential for identifying novel single-gene CNV associations, which could provide insights into the pathophysiology underlying neuropsychiatric disorders.

## Graphical abstract

Identification of neuropsychiatric CNVs. **A)** Schematic of a deletion and duplication. **B)** Example of a locus enriched for deletions in cases. **C)** Number of risk CNVs implicated in published studies of neuropsychiatric disorders. The number of cases included in each study is shown on the x axis, and larger points represent studies with more control samples (see Table 1 for further details). SCZ = schizophrenia, ASD = autism spectrum disorder, ID = intellectual disability, MDD = major depressive disorder, ADHD = attention-deficit hyperactivity disorder, TS = Tourette syndrome, OCD = obsessive compulsive disorder, BD = bipolar disorder.

**Figure:**
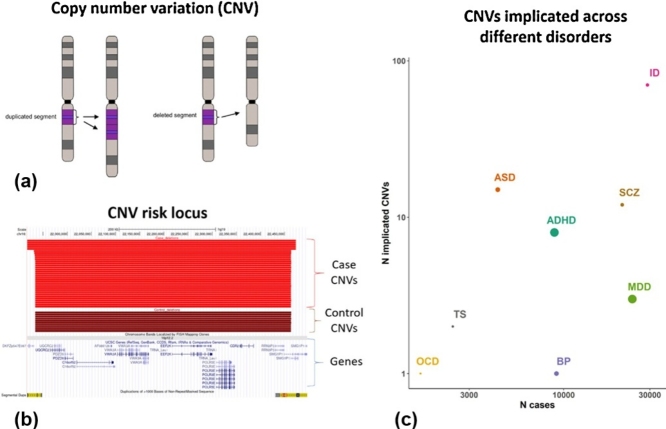

**Current Opinion in Genetics and Development** 2021, **68**:57–63

This review comes from a themed issue on **Molecular and genetic basis of disease**

Edited by **Jennifer Gladys Mulle**, **Patrick F Sullivan** and **Jens Hjerling-Leffler**

For a complete overview see the Issue and the Editorial

Available online 19th March 2021

**https://doi.org/10.1016/j.gde.2021.02.014**

0959-437X/© 2021 The Authors. Published by Elsevier Ltd. This is an open access article under the CC BY license (http://creativecommons.org/licenses/by/4.0/).

## Introduction

Copy number variants (CNVs) are structural rearrangements to chromosomes and represent a major source of genetic variation [1,2^••^]. CNVs involve gains and losses of DNA segments, respectively known as duplications and deletions. Other forms of structural variants include inversions, which reverse the sequence of a DNA segment, complex structural variants that involve repeated chromosomal rearrangements within the same locus (e.g. inversions within a duplication), and multi-allelic CNVs [2^••^]. CNVs are widely distributed across the genome, collectively accounting for more base-pair changes between individuals than all SNPs combined [3], and each event can affect between 1KB and several MB of DNA. Although common CNVs (e.g. those >1% in frequency) often represent benign polymorphisms, rare CNVs at specific loci have long been established as important risk factors for Mendelian disorders and complex neuropsychiatric disorders.

Since 22q11.2 deletions were first associated with increased risk for schizophrenia [4], large-scale genetic studies have identified additional CNVs that contribute to liability for different neuropsychiatric disorders, such as schizophrenia, autism spectrum disorder (ASD), intellectual disability (ID)/developmental delay (DD), major depressive disorder (MDD), attention-deficit hyperactivity disorder (ADHD), Tourette syndrome (TS) and obsessive compulsive disorder (OCD) (Table 1). Moreover, it has become increasingly apparent that the same CNV can increase risk for different disorders (i.e. they are pleiotropic) and that people without a diagnosed psychiatric disorder often carry risk CNVs (i.e. their penetrance is incomplete). However, despite these advances, understanding disease biology from CNV associations has been challenging as most risk CNVs are large and disrupt multiple genes, and the causal gene(s) underlying each CNV is not usually known. Nevertheless, animal and cellular models based on neuropsychiatric CNVs, as well CNV gene-set analyses, have provided important insights into the disease mechanisms underlying neuropsychiatric disorders.

**Table 1:** Copy number variant studies in neuropsychiatric disorders. The largest case-control and/or family studies that have analysed CNVs are presented for different neuropsychiatric disorders. The number of specific CNVs implicated across these studies is presented. SCZ = schizophrenia, ASD = autism spectrum disorder, ID = intellectual disability, DD = developmental delay, MDD = major depressive disorder, ADHD = attention-deficit hyperactivity disorder, TS = Tourette syndrome, OCD = obsessive compulsive disorder, BD = bipolar disorder

Phenotype	Cases	Controls	Trios/Quads	N implicated CNVs	Key references
SCZ	21 094	26 628	662	12	[5, 6, 7]
BD	9129	63 068		1	[8]
ADHD	8883	180 776	305	8	[9^•^,^10^]
ASD	4315	70 739	5574	15	[11, 12, 13]
MDD	23 979	383 095		3	[14^•^]
OCD	1613	1789	174	1	[15,16]
TS	2434	4093		2	[17]
ID/DD	29 085	19 584		70	[18]

In this review, we discuss the current understanding of CNVs that contribute to liability for neuropsychiatric disorders. We will describe the etiologic overlap between different psychiatric and neurodevelopmental disorders and recent findings from studies that have investigated the effects of neuropsychiatric CNVs in large population-based cohorts. Finally, we will address what these findings imply about the biological underpinnings of neuropsychiatric disorders.

## Neuropsychiatric CNV loci

A large body of evidence has shown rare CNVs contribute to the liability of most neuropsychiatric disorders, with the largest number of specific CNVs currently implicated in studies of ID/DD, ASD and schizophrenia (Table 1). In DD, case-control studies have found evidence for up to 70 associated CNVs [18], the majority of which are known causes of genomic disorders and occur in DNA regions prone to rearrangement via non-allelic homologous recombination [19]. In ASD, 15 CNVs across 10 loci have been implicated [11, 12, 13], whereas for schizophrenia strong statistical evidence has been found for up to 12 risk CNVs [5,6,20]. CNV burden analyses suggest that additional risk loci for schizophrenia will be discovered when larger samples become available [5], but their frequencies are likely to be lower than those observed for currently implicated CNVs. The frequencies and association statistics for selected neuropsychiatric CNVs and disorders as taken from published case-control studies are presented in Supplementary Table S1.

More recently, a role for rare CNVs in ADHD liability was confirmed in a large case-control sample from Iceland and Norway, where an excess burden of 19 known neuropsychiatric CNVs was found in cases (odds ratio (OR) (95% CI) = 2.43 (2.05, 2.87)), as well as significant association for 8 specific loci (Supplementary Table S1) [9^•^]. The rate of *de novo* CNVs in ADHD also appears to be greater than that observed in controls [10], with similar observations previously made for *de novo* CNVs in studies of schizophrenia [7,21] and ASD [13].

Rare pathogenic CNVs have also been shown to increase liability to more common mental health disorders, such as MDD. Here, short deletions (<100 kb) and known neuropsychiatric CNVs are enriched in people with depression [14^•^,^22^], and significant association has been found for 3 specific CNVs (1q21.1 duplication, PWS/AS duplication, and 16p11.2 duplication) (Supplementary Table S1) [14^•^].

The contribution of CNVs to BD liability is less clear; early reports implicated 1q21.1 duplications, 3q29 deletions and 16p11.2 duplications, but no significant difference was found for the genome-wide rate of CNVs in BD compared with controls [8]. Although current statistical evidence for specific risk CNVs in BD is weak, some CNVs have similar effect sizes to those observed in MDD (Supplementary Table S1), suggesting that larger samples may identify robust associations. However, the lack of robust CNV associations in BD could also, in part, be due to the less severe impairments in cognitive function that are found in BD patients when compared with other psychiatric disorders, such as schizophrenia [23,24], given neuropsychiatric CNVs have been associated with reduced cognitive function in the general population [25]. In support of this hypothesis, a recent study evaluated CNVs across different BD subphenotypes and found a significant enrichment of CNVs only in schizoaffective bipolar (SAB) cases compared to controls [26^•^], and patients diagnosed with SAB have greater cognitive impairments compared with patients with a diagnosis of BD type I or II [23]. Studies of common alleles in BD have also found evidence for genetic heterogeneity across BD subphenotypes, where significant elevations of schizophrenia PRS are found in SAB compared with other BD subphenotypes [27,28]. These findings highlight the importance of considering different genetic architectures across psychiatric subphenotypes when examining CNV associations.

Current CNV studies of TS and OCD are smaller than those conducted for DD, ASD and schizophrenia, although CNVs have also been shown to increase risk for these disorders [15, 16, 17,29], with deletions of *NRXN1* and duplications of *CNTN6* currently implicated in TS [17] and 16p13.11 deletions in OCD [15]. When tested as a group, the rate of neuropsychiatric CNVs is higher in TS/OCD compared with controls [15,17], which again suggests that additional specific neuropsychiatric CNVs will be associated with TS/OCD when evaluated in larger samples.

## Shared and unique effects of neuropsychiatric CNVs

**Figure 1:**
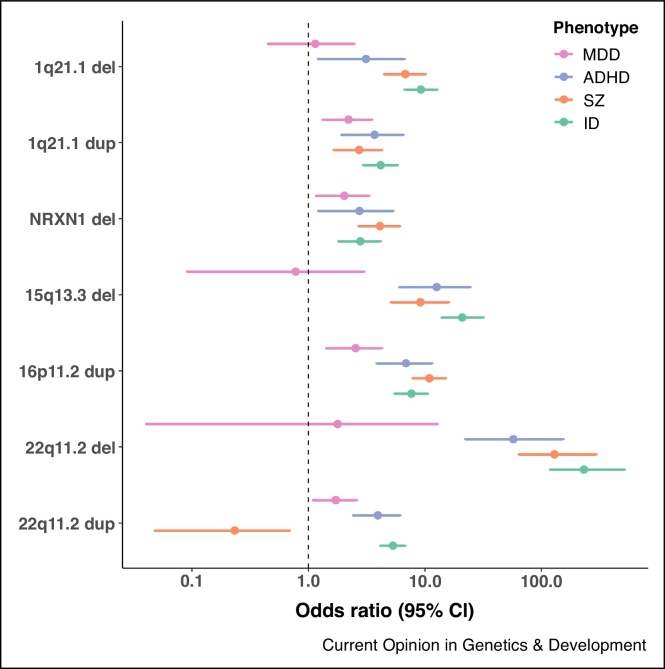
Odds ratios for selected neuropsychiatric CNVs across different disorders. To allow for a meaningful comparison of odds ratios across disorders, the CNV rate for each disorder was compared with the control rate reported in Kendall *et al.*, which has the largest number of controls [14^•^] (Supplementary Table S1).

As the number of established CNV loci for psychiatric and neurodevelopmental disorders has grown, it has become increasingly apparent that most neuropsychiatric CNVs confer risks for multiple disorders (Supplementary Table S1 and Figure 1). All known schizophrenia risk CNVs have been implicated in ID [6], and most CNVs implicated in ASD overlap those found in schizophrenia [30]. However, the penetrance of neuropsychiatric CNVs differs across different disorders, with carriers more likely to develop early-onset neurodevelopmental disorders such as ID/DD and ASD [31].

These shared CNVs point towards pleiotropic effects, which suggests that different neuropsychiatric disorders have overlapping disease biology; however, for multigenic CNVs it is not yet known whether the same genes are associated with different disorders [32], although the most parsimonious explanation is that any brain expressed gene in the CNV will have some effect on all neuropsychiatric disorders. Large-scale sequencing efforts may enable the causal gene(s) for multigenic CNVs to be identified, as genes associated with rare coding variants in neurodevelopmental disorders are enriched within neuropsychiatric CNV loci [33]. The only single gene CNV that is unequivocally associated with multiple neuropsychiatric disorders involve non-recurrent exonic deletions of *NRXN1*, indicating this gene has true pleiotropic effects. The most common phenotype associated with *NRXN1* deletions in clinical samples is ID [34], although schizophrenia, ASD and TS are also associated. Deletions affecting the promotor or exons at the 5’ end of *NRXN1* show higher penetrance for ID/DD compared with deletions only affecting the 3’ end [35], suggesting the size and location of *NRXN1* deletions might influence the highly variable phenotypic outcomes.

Accumulating evidence indicates that additional genetic variation modulates the phenotype expressed in carriers of specific neuropsychiatric CNVs. For example, individuals with a neuropsychiatric CNV and schizophrenia have an elevated burden of common schizophrenia risk alleles compared with controls [36,37]. Moreover, for individuals with a 22q11.2 deletion, higher schizophrenia polygenic risk scores and an elevated burden of rare loss-of-function variants in synaptic genes are found for carriers who have psychosis [38], whereas carriers who have ID have a higher burden of additional rare CNVs [39]. Similar findings have also been reported for individuals with ASD, where patients carrying multiple neuropsychiatric CNVs, or a specific neuropsychiatric CNV in addition to rare deleterious coding variants, have more severe neurodevelopmental phenotypes or comorbid ID [40^•^].

Identifying CNVs that discriminate between neuropsychiatric disorders is challenging due to differences in sample size across studies. However, one clear distinction is found for 22q11.2 duplications, which are known risk factors for ID/DD/ASD but have a significantly lower rate in schizophrenia compared with controls [5,30,41,42] (Supplementary Table S1 and Figure 1). Overall, the current evidence suggests that most, if not all, neuropsychiatric CNVs identified to date increase risk for multiple disorders.

## Neuropsychiatric CNVs in the general population

The development of large population based genetic cohorts, such as the UK Biobank, has enabled the effects of neuropsychiatric CNVs to be studied in individuals without a diagnosed neuropsychiatric disorder. The findings from these studies have shown that neuropsychiatric CNVs impact a wide range of cognitive, physical and medical traits. For example, data from the UK Biobank has indicated that nearly all neuropsychiatric CNVs are associated with reduced cognitive performance in people unaffected by psychiatric or neurodevelopmental phenotypes [43]. Here, the impact of specific CNVs on cognition was strongly correlated with their penetrance for developing a neurodevelopmental disorder (Pearson’s correlation = 0.74) [43]. These findings support a previous Icelandic study that found cognitive performance scores in unaffected CNV carriers were between those observed in schizophrenia patients and the general population [25].

Neuropsychiatric CNVs have also been shown to increase risk for a large number of non-psychiatric medical morbidities, such as diabetes, hypertension, cardiac, respiratory and renal disorders [44^•^], as well as physical traits (e.g. BMI, waist/hip ratio) [45]. However, some of these associations are likely to result from secondary effects (e.g. obesity leading to hypertension).

Large population-based cohorts have enabled the prevalence and penetrance estimates of neuropsychiatric CNVs to be refined, as they can sometimes overcome ascertainment biases that are inherent in case-control studies. For example, a recent study found 31 neuropsychiatric CNVs had a prevalence rate of 0.8% in a health care system–based population (90 595 individuals), and that carriers of these CNVs had increased rates of neuropsychiatric disorders (including common conditions such as depression and anxiety) and congenital malformations (the most common of which were cardiac defects) [46^••^]. Only 5.8% of individuals that carried a neuropsychiatric CNV had a documented genetic diagnosis, which supports the inclusion of neuropsychiatric disorders in future genomic screening programs [46^••^].

## Biological insights

One of the main goals of psychiatric genetics research is to advance our understanding of disease mechanisms and enable the development of new and more effective treatments. Indeed, early trials that tailored therapies towards biological systems hypothesised to be perturbed by specific CNVs in patients with psychotic disorders have shown promise for improving clinical symptoms [47]. However, it has been challenging to directly derive biological insights from large neuropsychiatric CNVs, as multiple genes within the locus are likely to have role [11,40^•^], and which specific genes are associated remains unknown. The exception is for single-gene CNVs that disrupt *NRXN1*, which encodes a synaptic neuronal adhesion molecule that is essential for synaptic formation, organisation and plasticity [48]. Association between non-recurrent *NRXN1* deletions and numerous psychiatric disorders (Supplementary Table S1) indeed implies shared disease mechanisms; however there are over 100 *NRXN1* isoforms, and heterogeneity in deletion size and location across patients [35,49] may contribute to variation in disease mechanisms associated with this locus.

CNV gene-set analyses have identified specific biological processes disrupted in neuropsychiatric disorders. For example, genes related to the activity-regulated cytoskeleton-associated protein and *N*-Methyl-D-aspartic acid synaptic complexes, as well as GABAergic and glutamatergic signalling and voltage-gated calcium channels, are associated with CNVs in schizophrenia [5,7,50,51]. Small *de novo* CNVs in ASD have also implicated networks of genes involved in chromatin regulation or synaptic proteins [13], showing convergence with findings from studies of schizophrenia.

Insights into the underlying pathology of neuropsychiatric disorders have also been gained from animal models of CNVs, which have recapitulated phenotypes observed in psychiatric disorders, such as impairments in cognition and social behaviour [52]. Neuronal phenotypes observed in CNV models include abnormal dopamine cell firing activity for 1q21.1 deletions [53], reduced excitatory synaptic transmission and synapse number for *NRXN1* deletions [54,55], and altered synaptic plasticity for 22q11.2 deletions [52]. Although different neuropsychiatric CNVs will directly impact distinct sets of genes, transcriptomic data from 15q13.3 deletion, 22q11.2 deletion, and 1q21.1 deletion mouse models has provided evidence that common modules of co-expressed genes are dysregulated by all three CNVs, suggesting the effects from different CNVs can converge on similar biological processes [56]. Additionally, studies involving human-induced pluripotent stem cells (iPSCs) derived from patients carrying neuropsychiatric CNVs have pointed towards abnormalities in dendritic morphology, reduced neuronal size and reduced synaptic density (see Ref. [57] for a detailed review on iPSCs and CNVs).

## Conclusion

Rare CNVs have been shown to be important risk factors for schizophrenia, ASD and ID. Through the development of large case-control and population based genetic datasets, CNVs have also more recently been shown to contribute to risk for ADHD and MDD. All established neuropsychiatric CNVs have substantial pleiotropic effects, which implies that some disorders in part have overlapping aetiologies. Longitudinal and systematic studies of specific neuropsychiatric CNVs have enabled a better understanding of the full spectrum of associated phenotypes, as well as their effects on individuals who have not developed a psychiatric or neurodevelopmental disorder. As most known neuropsychiatric CNVs disrupt multiple genes, it has been challenging to map these genetic associations to specific biological processes. However, a large population-based reference map of structural variants recently generated from whole-genome sequencing data, known as gnomAD-SV, has shown most structural variants carried by individuals are far too small to be reliably detected from conventional microarray data [2^••^]. Therefore, there are major gaps in our understanding of the contribution of small structural variants to the liability of psychiatric disorders. This is an important gap in knowledge to address, as smaller structural variants are more likely to disrupt individual genes, which may provide clearer mechanistic insights into disease biology. As previously shown in psychiatric CNV studies based on microarray data, extremely large whole-genome sequencing datasets will be required to identify robust statistical associations. Nevertheless, the gnomAD-SV data set will long serve as a critical resource for facilitating the discovery and interpretation of smaller neuropsychiatric structural variants in future studies.

## Conflict of interest statement

Nothing declared.

## References and recommended reading

Papers of particular interest, published within the period of review, have been highlighted as:

• of special interest
•• of outstanding interest
