# Supplementary material for: Copy number variation and neuropsychiatric illness
Source: Curr Opin Genet Dev. 2021 Jun;68:57–63. doi: 10.1016/j.gde.2021.02.014 (PMC8219524; doi:10.1016/j.gde.2021.02.014)
Supplement: Supplementary file 1 [file mmc1.docx]

**Supplementary Material**

**Table S1.** Neuropsychiatric CNV association statistics across multiple disorders. CNV frequencies, odds ratios and P values were taken from published studies that had more than 5,000 cases. Therefore, data from ASD, TS and OCD are not included in this table. Additionally, many loci implicated in autism studies are based on *de novo* CNVs, which is not appropriate for this table. Where odds ratios and P values are absent (indicated by “-“), the cited study did not report these statistics. We note that it is not possible to estimate odds ratios for CNVs with zero observations in controls, which is why these CNVs have been given “NA” for their odds ratio. SCZ = schizophrenia, ID = intellectual disability, MDD = major depressive disorder, ADHD = attention-deficit hyperactivity disorder, BD = bipolar disorder, del = deletion, dup = duplication, PWS/AS = Prader-Willi/Angelman syndrome = PWS/AS, Williams-Beuren syndrome = WBS.

| CNV  (Coordinates (Mb)) | Phenotype | Reference | CNV Frequency (%) | | Odds ratio (95% CI) | P |
| --- | --- | --- | --- | --- | --- | --- |
|  |  |  | Cases | Controls |  |  |
| 1q21.1 del  (chr1:146.53-147.39) | ADHD | [1] | 0.079 | 0.029 | 2.68 (0.92, 6.44) | 0.054 |
|  | BD | [2] | 0.033 | 0.021 | 1.61 (0.47, 5.5) | 0.44 |
|  | ID | [3] | 0.230 | 0.031 | 7.64 (3.33, 21.57) | 5.50E-10 |
|  | MDD | [4] | 0.029 | 0.026 | 1.11 (0.46, 2.22) | 0.79 |
|  | SCZ | [5] | 0.172 | 0.026 | 6.8 (2.9, 18.51) | 2.10E-07 |
| 1q21.1 dup  (chr1:146.53-147.39) | ADHD | [1] | 0.146 | 0.045 | 3.44 (1.67, 6.52) | 0.002 |
|  | BD | [2] | 0.099 | 0.037 | 2.64 (1.19, 5.88) | 0.022 |
|  | ID | [3] | 0.170 | 0.026 | 6.47 (2.59, 20.84) | 6.50E-07 |
|  | MDD | [4] | 0.088 | 0.040 | 2.17 (1.34, 3.36) | 9.10E-04 |
|  | SCZ | [5] | 0.108 | 0.049 | 2.3 (1.08, 5.09) | 0.022 |
| *NRXN1* del  (chr2:50.15-51.26) | ADHD | [1] | 0.101 | 0.021 | 4.68 (1.82, 10.64) | 0.0026 |
|  | BD | [2] | 0.000 | 0.020 | - | 1 |
|  | ID | [3] | 0.103 | 0.046 | 2.25 (1.04, 5.38) | 0.00005 |
|  | MDD | [4] | 0.075 | 0.037 | 2.01 (1.18, 3.19) | 5.70E-03 |
|  | SCZ | [5] | 0.152 | 0.034 | 4.5 (2.03, 10.94) | 0.000028 |
| 3q29 del  (chr3:195.72-197.35) | ADHD | [1] | 0.011 | 0.002 | - |  |
|  | BD | [2] | 0.025 | 0.001 | 17.31 (1.57, 190.97) | 0.03 |
|  | ID | [3] | 0.038 | 0.000 | NA | 0.0035 |
|  | MDD | [4] | 0.013 | 0.001 | 11.22 (2.27, 46.52) | 1.00E-03 |
|  | SCZ | [5] | 0.069 | 0.004 | 18 (2.66, 763.34) | 0.0002 |
| WBS dup  (chr7:72.74-74.14) | ADHD | [1] | 0.011 | 0.002 | - |  |
|  | BD | [2] | 0.000 | 0.006 | - | 1 |
|  | ID | [3] | 0.096 | 0.000 | NA | 5.46E-07 |
|  | MDD | [4] | 0.0083 | 0.001 | - |  |
|  | SCZ | [5] | 0.039 | 0.008 | 5.2 (1, 51.55) | 0.045 |
| 15q11.2 del  (chr15:22.81-23.09) | ADHD | [1] | 0.417 | 0.244 | 1.65 (1.11, 2.37) | 0.016 |
|  | BD | [2] | 0.170 | 0.280 | 0.6 (0.36, 1.02) | 0.052 |
|  | ID | [3] | 0.688 | 0.138 | 5.06 (3.34, 7.80) | 3.19E-21 |
|  | MDD | [4] | 0.42 | 0.386 | 1.11 (0.9, 1.35) | 3.00E-01 |
|  | SCZ | [5] | 0.642 | 0.368 | 1.8 (1.35, 2.38) | 0.000023 |
| PWS/AS dup  (chr15:22.81-28.39) | ADHD | [1] | 0.000 | 0.009 | - |  |
|  | BD | [2] | 0.000 | 0.006 | - | 1 |
|  | ID | [3] | 0.165 | 0.000 | NA | 1.82E-11 |
|  | MDD | [4] | 0.025 | 0.003 | 8.14 (2.77, 21.69) | 4.61E-05 |
|  | SCZ | [5] | 0.083 | 0.000 | NA | 4E-07 |
| 15q13.3 del  (chr15:31.08-32.46) | ADHD | [1] | 0.135 | 0.019 | 5.97 (2.63, 12.6) | 0.0001 |
|  | BD | [2] | 0.043 | 0.019 | 1.32 (0.3, 5.8) | 0.66 |
|  | ID | [3] | 0.223 | 0.000 | NA | 2.85E-15 |
|  | MDD | [4] | 0.0083 | 0.011 | 0.77 (0.13, 2.52) | 7.20E-01 |
|  | SCZ | [5] | 0.098 | 0.019 | 4.6 (1.64, 16.22) | 0.0015 |
| 16p13.11 dup  (chr16:15.51-16.29) | ADHD | [1] | 0.293 | 0.129 | 2.12 (1.31, 3.27) | 0.0035 |
|  | BD | [2] | 0.110 | 0.130 | 0.83 (0.42, 1.64) | 0.75 |
|  | ID | [3] | 0.234 | 0.138 | 1.70 (1.07, 2.76) | 0.0112 |
|  | MDD | [4] | 0.017 | 0.196 | 0.87 (0.63, 1.78) | 3.90E-01 |
|  | SCZ | [5] | 0.377 | 0.222 | 1.7 (1.2, 2.52) | 0.0022 |
| 16p12.1 del  (chr16:21.95-22.43) | ADHD | [1] | 0.101 | 0.066 | 1.52 (0.63, 3.16) | 0.26 |
|  | BD | [2] | - | - | - | - |
|  | ID | [3] | 0.172 | 0.056 | 3.06 (1.57, 6.53) | 1.77E-04 |
|  | MDD | [4] | 0.083 | 0.056 | 1.47 (0.9, 2.27) | 9.00E-02 |
|  | SCZ | [5] | 0.162 | 0.045 | 3.3 (1.61, 7.05) | 0.00034 |
| 16p11.2 distal del  (chr16:28.82-29.04) | ADHD | [1] | 0.034 | 0.018 | 2.19 (0.42, 7.29) | 0.19 |
|  | BD | [2] | - | - | - | - |
|  | ID | [3] | 0.093 | 0.005 | 18.20 (3.00, 742.63) | 1.09E-05 |
|  | MDD | [4] | 0.029 | 0.013 | 2.23 (0.92, 4.63) | 5.00E-02 |
|  | SCZ | [5] | 0.025 | 0.019 | 1.7 (0.37, 7.6) | 0.51 |
| 16p11.2 del  (chr16:29.65-30.20) | ADHD | [1] | 0.079 | 0.035 | 2.16 (0.75, 5.11) | 0.14 |
|  | BD | [2] | - | - | - | - |
|  | ID | [3] | 0.347 | 0.031 | 11.37 (5.04, 31.74) | 2.07E-16 |
|  | MDD | [4] | 0.033 | 0.027 | 1.21 (0.54, 2.34) | 6.00E-01 |
|  | SCZ | [5] | 0.029 | 0.049 | 0.61 (0.19, 1.79) | 0.47 |
| 16p11.2 dup  (chr16:29.65-30.20) | ADHD | [1] | 0.191 | 0.044 | 4.34 (2.27, 7.81) | 0.000091 |
|  | BD | [2] | 0.130 | 0.030 | 4.37 (2.12, 9) | 0.00023 |
|  | ID | [3] | 0.213 | 0.046 | 4.65 (2.30, 10.64) | 3.50E-07 |
|  | MDD | [4] | 0.071 | 0.028 | 2.65 (1.53, 4.31) | 2.04E-04 |
|  | SCZ | [5] | 0.304 | 0.030 | 11 (5.08, 26.43) | 3.70E-15 |
| 22q11.2 del  (chr22:19.04-21.47) | ADHD | [1] | 0.135 | 0.016 | 10.73 (4.66, 23.15) | 1.8E-06 |
|  | BD | [2] | 0.012 | 0.000 | NA | 0.095 |
|  | ID | [3] | 0.543 | 0.000 | NA | 3.97E-36 |
|  | MDD | [4] | 0.0042 | 0.002 | 1.69 (0.09, 9.17) | 6.20E-01 |
|  | SCZ | [6] | 0.303 | 0.0049 | 67.7 (9.3 – 492.8) | 5.70E-18 |
| 22q11.2 dup  (chr22:19.04-21.47) | ADHD | [1] | 0.248 | 0.100 | 2.24 (1.32, 3.63) | 0.0042 |
|  | BD | [2] | - | - | - | - |
|  | ID | [3] | 0.334 | 0.061 | 5.46 (2.99, 10.93) | 1.35E-11 |
|  | MDD | [4] | 0.011 | 0.063 | 1.72 (1.12, 2.53) | 9.00E-03 |
|  | SCZ | [5] | 0.015 | 0.079 | 0.2 (0.038, 0.71) | 0.0046 |

**References**

1. Gudmundsson OO, Walters GB, Ingason A, Johansson S, Zayats T, Athanasiu L, Sonderby IE, Gustafsson O, Nawaz MS, Jonsson GF, et al.: **Attention-deficit hyperactivity disorder shares copy number variant risk with schizophrenia and autism spectrum disorder**. *Transl Psychiatry* 2019, **9**:258.

2. Green EK, Rees E, Walters JTR, Smith KG, Forty L, Grozeva D, Moran JL, Sklar P, Ripke S, Chambert KD, et al.: **Copy number variation in bipolar disorder**. *Mol Psychiatry* 2016, **21**:89–93.

3. Coe BP, Witherspoon K, Rosenfeld JA, van Bon BWM, Vulto-van Silfhout AT, Bosco P, Friend KL, Baker C, Buono S, Vissers LELM, et al.: **Refining analyses of copy number variation identifies specific genes associated with developmental delay**. *Nat Genet* 2014, **46**:1063–1071.

4. Kendall KM, Rees E, Bracher-Smith M, Legge S, Riglin L, Zammit S, O’Donovan MC, Owen MJ, Jones I, Kirov G, et al.: **Association of Rare Copy Number Variants With Risk of Depression**. *JAMA Psychiatry* 2019, **76**:818–825.

5. Rees E, Kendall K, Pardiñas AF, Legge SE, Pocklington A, Escott-Price V, MacCabe JH, Collier DA, Holmans P, O’Donovan MC, et al.: **Analysis of intellectual disability copy number variants for association with schizophrenia**. *JAMA Psychiatry* 2016, **73**:963–969.

6. Marshall CR, Howrigan DP, Merico D, Thiruvahindrapuram B, Wu W, Greer DS, Antaki D, Shetty A, Holmans PA, Pinto D, et al.: **Contribution of copy number variants to schizophrenia from a genome-wide study of 41,321 subjects**. *Nature Genetics* 2017, **49**:27–35.
